# Genome Rearrangement Shapes Prochlorococcus Ecological Adaptation

**DOI:** 10.1128/AEM.01178-18

**Published:** 2018-08-17

**Authors:** Wei Yan, Shuzhen Wei, Qiong Wang, Xilin Xiao, Qinglu Zeng, Nianzhi Jiao, Rui Zhang

**Affiliations:** a State Key Laboratory of Marine Environmental Science, College of Ocean and Earth Sciences, Institute of Marine Microbes and Ecospheres, Xiamen University, Xiamen, People's Republic of China; b Division of Life Science, the Hong Kong University of Science and Technology, Clear Water Bay, Hong Kong, People's Republic of China; c Department of Ocean Science, the Hong Kong University of Science and Technology, Clear Water Bay, Hong Kong, People's Republic of China; McMaster University

**Keywords:** Prochlorococcus, genome evolution, genome rearrangement, genomic backbone

## Abstract

Prochlorococcus is the most abundant and smallest known free-living photosynthetic microorganism and is a key player in marine ecosystems and biogeochemical cycles. Prochlorococcus can be broadly divided into high-light-adapted (HL) and low-light-adapted (LL) clades. In this study, we isolated two low-light-adapted clade I (LLI) strains from the western Pacific Ocean and obtained their genomic data. We reconstructed Prochlorococcus evolution based on genome rearrangement. Our results showed that genome rearrangement might have played an important role in Prochlorococcus evolution. We also found that the Prochlorococcus clades with streamlined genomes maintained relatively high synteny throughout most of their genomes, and several regions served as rearrangement hotspots. Backbone analysis showed that different clades shared a conserved backbone but also had clade-specific regions, and the genes in these regions were associated with ecological adaptations.

**IMPORTANCE** Prochlorococcus, the most abundant and smallest known free-living photosynthetic microorganism, plays a key role in marine ecosystems and biogeochemical cycles. Prochlorococcus genome evolution is a fundamental issue related to how Prochlorococcus clades adapted to different ecological niches. Recent studies revealed that the gene gain and loss is crucial to the clade differentiation. The significance of our research is that we interpreted the Prochlorococcus genome evolution from the perspective of genome structure and associated the genome rearrangement with the Prochlorococcus clade differentiation and subsequent ecological adaptation.

## Abstract (precis)

Prochlorococcus, the most abundant and smallest known free-living photosynthetic microorganism, plays a key role in marine ecosystems and biogeochemical cycles. Prochlorococcus genome evolution is a fundamental issue related to how Prochlorococcus clades adapted to different ecological niches. Recent studies revealed that the gene gain and loss is crucial to the clade differentiation. The significance of our research is that we interpreted the Prochlorococcus genome evolution from the perspective of genome structure and associated the genome rearrangement with the Prochlorococcus clade differentiation and subsequent ecological adaptation.

## INTRODUCTION

Prochlorococcus, a marine cyanobacterium, is the most abundant and smallest known free-living photosynthetic microorganism (1–3). Prochlorococcus is mainly distributed in the euphotic zone of nutrient-poor tropical and subtropical waters and is a key player in marine ecosystems and biogeochemical cycles (2, 4, 5). With a cell concentration of up to 10^5^ cells/ml in the oligotrophic surface ocean, Prochlorococcus accounts for 40% to 60% of photosynthetic biomass and produces approximately 4 gigatons of fixed carbon each year (2, 6–10).

In recent decades, at least 12 different Prochlorococcus clades were discovered in the world's oceans, which can be broadly divided into two major groups, high-light-adapted (HL) and low-light-adapted (LL) groups (11, 12), but only five clades have been cultivated: HLI, HLII, LLI, LLII/III, and LLIV (13–19). With the rapid development of sequencing technologies, dozens of Prochlorococcus genomes were published (20–25). The HL and LLII/III clades have genomes as small as 1.6 to 1.7 Mbp that encode 1,800 to 1,900 genes, which makes them the smallest known genomes among nonsymbiotic photosynthetic autotrophs (26). The LLI clade genomes are slightly larger (1.8 to 1.9 Mbp) and encode 2,100 to 2,200 genes. In contrast, the LLIV genome is approximately 2.4 to 2.6 Mbp, which is similar to that of the marine Synechococcus (24, 25).

Comparative genomics reveal that genome streamlining is a striking feature of Prochlorococcus genomes (22, 24, 27, 28). Highly compressed genomes allow Prochlorococcus to thrive in oligotrophic oceans by requiring less energy and fewer nutrients (3, 19, 24). The HL, LLI, and LLII/III clades, which have undergone substantial genome reduction compared with the LLIV clade and Synechococcus, have significantly different physiological characteristics, ecological distributions, and genomic content (3, 24). The HLI and HLII clades are distinguished by their temperature optima, account for most of the known Prochlorococcus, and are distributed in the upper euphotic zone (5). The LLI clade, which is usually found in the middle euphotic zone, has characteristics that are intermediate between HL and other LL clades (4, 5, 29). It is well known that the LLI clade contains more HL-inducible genes (*hli*), which encode proteins that protect cells from light shock as well as other stresses, and is the only LL clade that encodes photolyase, which is a photoprotective enzyme (19, 24). Currently, there are only four published genomes of this clade (24, 25). In contrast, the LLII/III clade, which was originally defined as two separate clades (LLII and LLIII) (13), is more restricted to the deep euphotic zone (4). In terms of genome reduction, one hypothesis is that early genome streamlining resulted in cell size reduction and facilitated light absorption of cells in low-light environments (3), and the LLII/III clade might diverge from this event. Previous studies also found that genome reduction was initiated when the most recent common ancestor (MRCA) of the HL, LLI, and LLII/III clades diverged from the nonstreamlined LLIV clade (24, 27). Thus, comparative genomic analysis of these streamlined but substantially different Prochlorococcus clades may provide insight into the key evolutionary processes associated with genome reduction, clade differentiation, and ecological adaptation.

Previous studies on Prochlorococcus genome evolution mainly focused on local mutations that only affected individual genes; for example, genomic content was compared between LL and HL genomes (22), hypervariable genomic islands (23), and gene gain/loss among different clades (24, 27). Those investigations reconstructed the history of vertical inheritance and horizontal gene transfer. In contrast to local mutations, genomes can also undergo large-scale mutation events, such as genome rearrangement, which can result in substantial changes in gene order and genomic content (30). Genome rearrangements are rare events and include inversions, deletions, duplications, and translocations (30, 31), and they have the potential to elucidate genome evolution (32, 33). In the case of Prochlorococcus, only a couple of studies were related to genome rearrangement (33, 34).

Here, we compared Prochlorococcus genomes, including two newly isolated LLI strains from the western Pacific Ocean. We reconstructed Prochlorococcus phylogeny based on genome rearrangement and showed that genome rearrangement might have played an important role in Prochlorococcus evolution. We also performed backbone analysis and showed that different clades shared a conserved backbone but also had clade-specific regions, and we revealed that the genes in these regions were associated with ecological adaptation.

## RESULTS AND DISCUSSION

### Genomes of the two newly isolated LLI strains.

In this study, two Prochlorococcus strains were obtained from the western Pacific Ocean, which is an undersampled region for Prochlorococcus. These two strains were both isolated at 150-m depths but from two different stations. XMU1408 was isolated from the Luzon strait, which is the main channel between the South China Sea and the western Pacific Ocean, whereas XMU1403 was isolated from open water of the western Pacific Ocean (detailed locations are described in Table 1). Preliminary internal transcribed spacer (ITS) analysis showed that these two strains belonged to the LLI clade, which was further confirmed by the phylogenomic analysis based on 31 core genes (see below). The completeness and contamination of the recovered genomes were estimated by CheckM (35). The unialgal status was confirmed by obtaining a single Prochlorococcus ITS sequence from the assembled contigs.

**TABLE 1:** Prochlorococcus strain isolation location, genome characteristics, and assembly statistics

Genome feature	Value for strain:	
	XMU1403	XMU1408
Ecotype/clade	LLI	LLI
Isolation location		
Longitude (E)	124	121
Latitude (N)	20	20
Isolation depth (m)	150	150
Sequencing method	HiSeq	HiSeq
Read length (bp)	150 × 2	150 × 2
Assembly size (bp)	1,746,033	1,795,147
G+C content (%)	35.2	34.1
No. of contigs	7	6
N_50_ (bp)	335,809	389,747
No. of protein-coding sequences	2,042	2,059
Completeness^*a*^ (%)	>98	>98
NCBI accession no.	QJUF00000000	QJUE00000000

a Completeness is estimated by CheckM (35).

Detailed genome analysis showed that XMU1403 and XMU1408 had genome sizes of 1.746 Mb (GC content, 35.2%) and 1.795 Mb (GC content, 34.1%), respectively, which contained 2,042 and 2,059 protein-coding sequences, respectively (Table 1). These genomic features were similar to those of the LLI strains isolated from other regions (see Table 3). Because there are only four LLI genomes published at present (24, 25) and a previous study found that LL strains contain a high number of novel genes (19), the genomic data of these first two LLI strains isolated from the western Pacific Ocean may enhance the understanding of the genetic diversity of this clade. Based on the six known LLI genomes, we calculated pan-, core, and accessory genome sizes of the LLI clade (2.74 Mb, 1.35 Mb, and 1.39 Mb, respectively). Clustering analysis of core and accessory genome fragments showed that each strain has its own unique accessory genome regions, and XMU1408 had greater differences than other strains (see Fig. S1 in the supplemental material), which is consistent with the results of the phylogenomic analysis (see below).

### Prochlorococcus phylogenomics.

**FIG 1:**
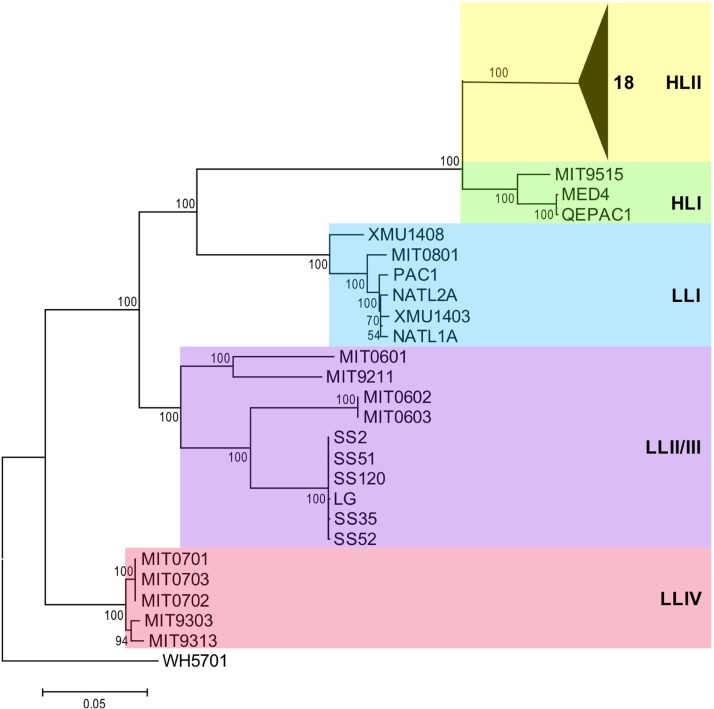
Phylogenomic relationships of Prochlorococcus. Shown is a phylogenetic tree reconstructed by concatenated protein sequences of 31 core genes with the maximum likelihood method, using Synechococcus sp. strain WH5701 as an outgroup. Numbers at the nodes represent bootstrap values (1,000 resamplings).

To reconstruct a robust phylogeny of Prochlorococcus for genomic study, we analyzed concatenated protein sequences of 31 core genes. The resulting phylogenetic tree, which is consistent with previous phylogenomic studies that used other methods (24, 28), showed that Prochlorococcus consists of at least five clades with relatively large phylogenetic distances. The HLI and HLII clades formed a monophyletic HL clade; in contrast, LL clades were more divergent (Fig. 1). It is also worth noting that the streamlined HL, LLI, and LLII/III clades shared a most recent common ancestor (MRCA), which had a large phylogenetic distance from the nonstreamlined LLIV clade. The LLI clade clustered with the HL clade, which is consistent with its intermediate-light adaptation. At the subclade level, XMU1408 was separated from other strains in the LLI clade, whereas the 10 LLII/III strains could also be divided into at least two subclades, which indicates that the LLI and LLII/III clades have complex evolutionary histories and different subclades.

### Genome rearrangement of streamlined clades.

**FIG 2:**
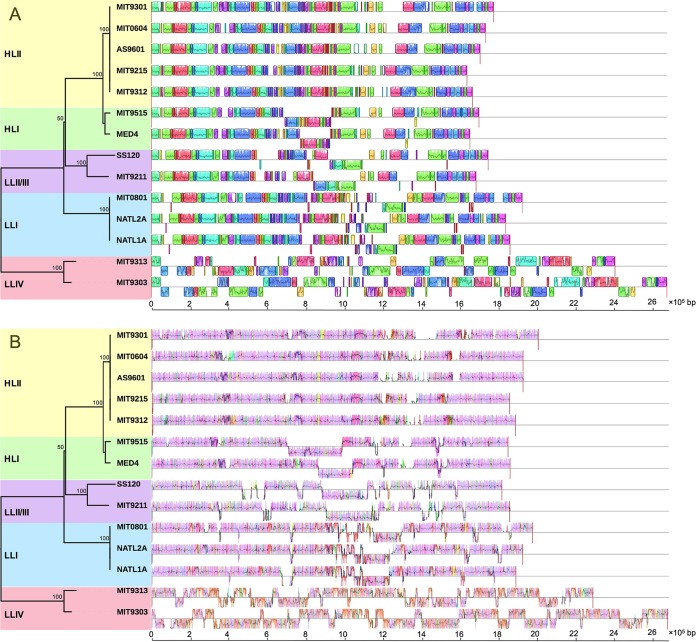
Genome comparison among 14 Prochlorococcus complete genomes. The genome comparison was generated by Mauve (53). (A) Rearrangements of locally colinear blocks (LCBs) among Prochlorococcus genomes. LCBs are color coded. LCBs below the black line have an inverse orientation relative to that of MIT9301. (B) Visualization of the backbone and clade-specific regions among Prochlorococcus genomes. Pink-colored segments are conserved among all strains (backbone), whereas other colored segments are conserved in a subset of strains. Segments below the black line have an inverse orientation relative to that of MIT9301. For both panels A and B, the large white regions indicate strain-specific content. The phylogenetic tree was reconstructed based on permutations of 69 LCBs with the maximum likelihood method. Numbers at the nodes represent bootstrap values (1,000 resamplings).

It is believed that genome streamlining is the result of Prochlorococcus adaptation to a relatively stable ocean surface environment (3, 19, 24). Comparative genomics of these streamlined but substantially different genomes may provide insight into the key evolutionary processes that shaped ecological adaptations of Prochlorococcus. In this study, we attempted to compare the Prochlorococcus genomes based on genome rearrangement. Sixty-nine LCBs were detected across 14 complete genomes of Prochlorococcus (Fig. 2A), which indicates a long and complex evolutionary history of Prochlorococcus. After excluding the LLIV clade, the number of LCBs of the three streamlined clades (HL, LLI, and LLII/III) was substantially reduced to 39, which signifies that the LLIV clade had a large phylogenetic distance from other clades. It is worth noting that most parts of the streamlined LLI, LLII/III, and HL genomes maintained relatively high synteny but substantially differed from the LLIV genomes (Fig. 2A and Fig. S2). Interestingly, the average LCB lengths of these streamlined clades were similar but significantly shorter than that of the LLIV clade (*P* < 0.001 by one-sample *t* test, *n* = 34; null hypothesis was set as 55,187 bp [the average LCB length of the LLIV clade]) (Fig. S3 to S7 and Table S1). These results indicate that the major genome rearrangement events coincided with genome reduction and occurred before divergence of the LLI, LLII/III, and HL clades. Previous studies on gene gain and loss also revealed that genome reduction occurred just after divergence of the LLIV clade and the MRCA of other clades (24, 27).

Phylogenomic analysis based on genome rearrangement reconstructs important evolutionary events of genome structure. In this study, the genome rearrangement-based phylogenomic tree showed a topology similar to that of the tree based on concatenated protein sequences of 31 core genes but with one discrepancy: the HL and LLII/III clades clustered into one group in the genome rearrangement-based tree (Fig. 2). This result is consistent with a previous study that reconstructed Prochlorococcus phylogeny based on gene order (33). It is possible that this discrepancy reflects a higher rate of genome rearrangement in the LLI clade. In detail, the LLII/III clade, which is usually found in deep waters and possesses more DNA repair genes (24), resulting in a low mutation rate, may have retained more of the genome structure of the MRCA of these streamlined clades; in contrast, the HL and LLI clades, which possess fewer DNA repair genes (24), could have less genome structure similarity because of a higher mutation rate of genome rearrangement in the middle to upper euphotic zone, although the latter two may share an MRCA and more similar genomic content. Our explanation does not contradict another hypothesis, which states that increase in mutation rate is the primary cause of genome reduction in Prochlorococcus (36), because the loss of many DNA repair genes in the streamlined HL and LLI genomes (24) could increase the local mutation rate and also the probability of genome rearrangement and thereby enhance Prochlorococcus clade differentiation.

Genome rearrangement is a major genomic mutation that can result from inversions, deletions, duplications, and translocations. Generally, these events are triggered by DNA double-helix breaks at two different sites, followed by rejoining of the broken ends that produce large changes in gene order and genomic content among closely related genomes. Genome rearrangement can result in destruction of gene structure when a breakpoint occurs inside a gene and can also have substantial influence on gene expression (30, 37, 38). Changes in genome structure are almost always destructive; however, in very rare cases, they provide significant benefits. In the case of Prochlorococcus evolution, the right genome rearrangement may provide significant advantages for occupying a new ecological niche (e.g., different light intensity and temperature), promoting clade differentiation and destroying dispensable genes, which results in genome reduction.

**FIG 3:**
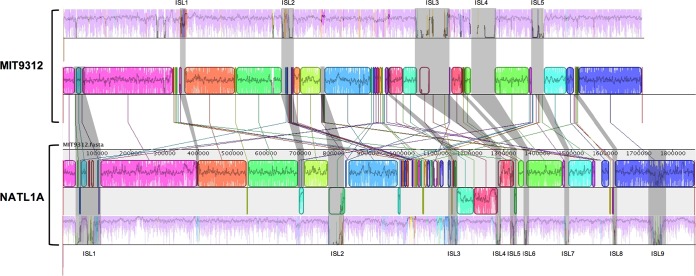
Comparison of the backbone, genomic islands, and locally colinear blocks (LCBs) between the HLII (MIT9312) and LLI (NATL1A) clades. The genome comparison was generated by Mauve (53). Pink-colored segments are conserved among all strains within a clade (backbone), and other colored segments are conserved in a subset of strains. Shaded regions indicate genomic islands (ISL). LCBs are color coded. LCBs below the black line have an inverse orientation relative to that of MIT9312. Connecting lines between the two genomes indicate corresponding LCBs.

To better elucidate genome rearrangement, we compared two complete genomes from the HLII clade (MIT9312) and the LLI clade (NATL1A) in detail. Based on genome alignment, nine major genomic islands were found in the LLI genomes (we define major genomic islands as highly nonconserved regions that appeared in at least half of the genomes within a clade) (Fig. 3). By comparing the HLII and LLI genomes, we found that most LCB rearrangements and clade-specific segments (see below) were located in the vicinity of the genomic islands (Fig. 3). For example, in the NATL1A genome, most LCBs that have an inverse orientation relative to MIT9312 (LCB inversions) locate at or near the genome islands (Fig. 3). Genomic islands 1 and 2 (ISL 1 and 2) in the MIT9312 genome have multiple rearrangements (LCB translocations, shown as LCB linking to different locations in NATL1A in Fig. 3). These results indicate that the genomic islands serve as hotspots that induce genome rearrangements.

### Genomic backbone and clade-specific segments.

**FIG 4:**
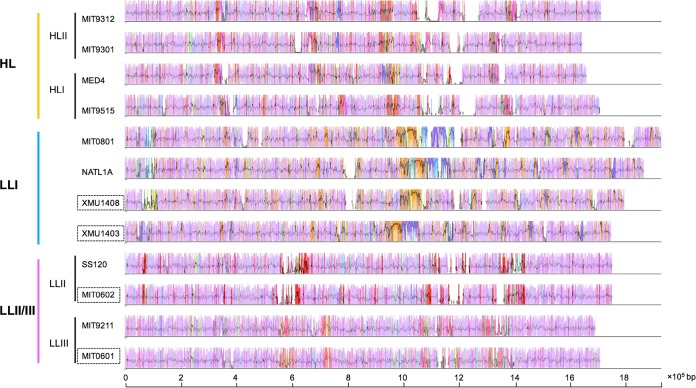
Visualization of the backbone and clade-specific regions among streamlined Prochlorococcus genomes. The genome comparison was generated by Mauve (53). Pink-colored segments are conserved among all strains (backbone), whereas segments in other colors are conserved in a subset of strains. All segments are above the black line because orientation was set relative to each genome for easier visualization. The large white regions indicate strain-specific content. Genome names in dashed boxes indicate draft genomes.

**FIG 5:**
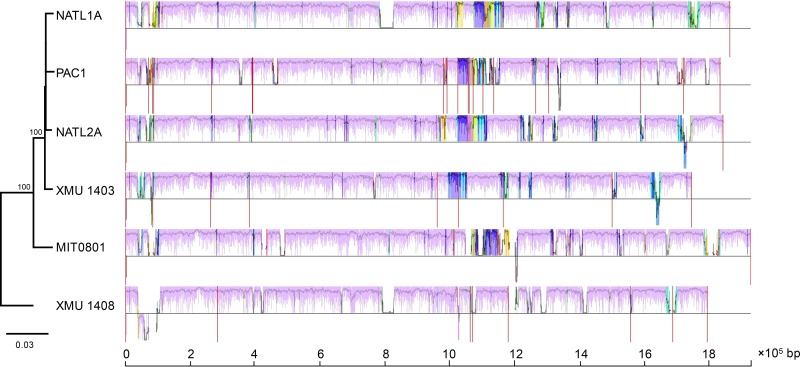
Visualization of the backbone and subclade-specific regions among six Prochlorococcus LLI clade genomes. The genome comparison was generated by Mauve (53). Pink-colored segments are conserved among all strains (backbone), whereas segments in other colors are conserved in a subset of strains. Segments below the black line have an inverse orientation relative to PAC1. The large white regions indicate strain-specific content. The phylogenetic tree was reconstructed using concatenated protein sequences of 31 core genes with the maximum likelihood method using MIT9313 as an outgroup (not shown). Numbers at the nodes represent bootstrap values (1,000 resamplings). Vertical red lines indicate contig breaks.

**FIG 6:**
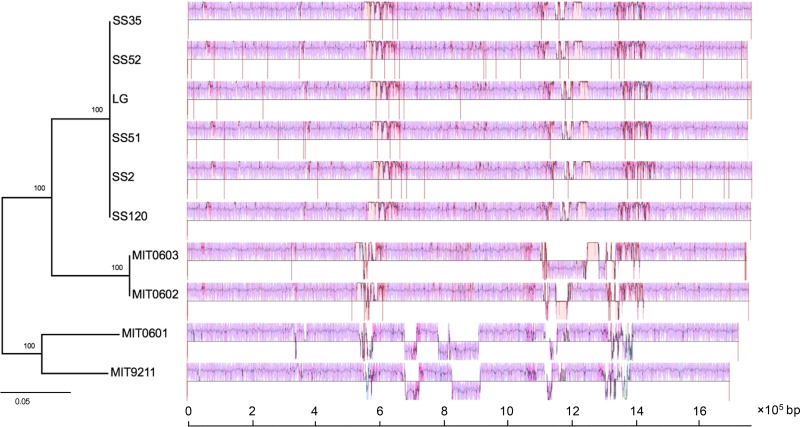
Visualization of the backbone and subclade-specific regions among 10 Prochlorococcus LLII/III clade genomes. The genome comparison was generated by Mauve (53). Pink-colored segments are conserved among all strains (backbone), whereas other segments are conserved in a subset of strains. Segments below the black line have an inverse orientation relative to that of SS120. The large white regions indicate strain-specific content. The phylogenetic tree was reconstructed using concatenated protein sequences of 31 core genes with the maximum likelihood method using MIT9313 as an outgroup (not shown). Numbers at the nodes represent bootstrap values (1,000 resamplings). Vertical red lines indicate contig breaks.

Comparison of closely related genomes has revealed the existence of highly conserved regions that form a genomic backbone, which is interrupted by many, less conserved genome regions (39). To investigate genome evolution, genome segmentation into backbone and variable regions is very useful. Thus, we performed genomic backbone analysis of streamlined clades and attempted to find the regions that may have served as hotspots of Prochlorococcus clade differentiation and ecological adaptation. The genome regions shared between all clades (backbones) and the regions shared between a subset of clades (clade-specific segments) were detected using genome alignment. Our results showed that the HL, LLI, and LLII/III genomes shared a conserved backbone, which was substantially different from that of LLIV clade (Fig. 2B), and each clade contained several clade-specific segments (Fig. 2B and 4). Compared to genome rearrangement, we found that these regions seemed to occur predominantly near regions that underwent genome rearrangement (Fig. 2B). In addition, we extended backbone analysis to the subclade level and found that there are substantial subclade-specific segments within clades. For example, XMU1408 has a different backbone pattern from other LLI strains (Fig. 5). In the LLII/III clade, the subclades also have different subclade-specific regions and genome rearrangement patterns (Fig. 6), which indicates that there are complex subclades within the LLII/III clade. A recent study grouped LLII and LLIII into a single clade (LLII/III) because they do not have a resolved phylogenetic relationship (19); however, our results indicate that LLII and LLIII belong to at least two separate clades based on genome structure differences (although, in this study, we still treated them as two subclades in the LLII/III clade for coherence).

### Genes in clade/subclade-specific segments.

Clade-specific segments are conserved regions shared within a clade but not with other clades; in contrast, genomic islands are hypervariable regions between different strains within a clade. Thus, the genes in clade-specific segments reflect clade differentiation and ecological adaptation, whereas genes in genomic islands reflect adaptations of individual strains to local niches. In this study, we attempted to find genes in these clade-specific regions and interpret the Prochlorococcus clade differentiation. Generally, most of the genes with known functions in clade-specific segments are related to light shock protection, DNA repair, and transporters, which correspond to the most dominant factors associated with Prochlorococcus evolution: light, DNA damage, and nutrient limitations (Table 2). At the clade level, the clade-specific segments of the LLI clade contain several genes that encode DNA ligase, high-light-induced proteins (HLIPs), and transporters. The unique regions of the HL clade have several genes for phosphorus transporters. It is worth noting that the HL and LLI clades share a segment that contains a urea assimilation gene cluster (although individual HL strains, such as MIT9515, lost this segment) (Table 2). The specific regions of the LLII/III clade contain genes for HLIP and DNA repair. We also extended this analysis to the subclade level. For example, compared to other LLI genomes, XMU1408 lacks a region that contains HLIP and DNA repair genes, while the LLII subclade has a region that contains DNA repair genes compared to the LLIII subclade (Table 2).

**TABLE 2:** Examples of genes found in clade-specific regions

Section	Gene name	Product	Locus
HL clade-specific region		Possible phosphate-binding protein	MED4_665181_665417
	*pstC*	Phosphate transport system permease protein PstC	MED4_685879_686826
	*pstA*	Phosphate transport system permease protein PstA	MED4_686833_687726
	*pstB*	Phosphate transport ATP-binding protein PstB	MED4_687728_688537
LLI clade-specific region	*pstB*	Phosphate transport ATP-binding protein PstB	NATL2A_847867_847064
	*pstA*	Phosphate transport system permease protein PstA	NATL2A_848813_847899
	*pstC*	Phosphate transport system permease protein PstC	NATL2A_849674_848814
		DNA ligase	NATL2A_955315_954713
		DNA ligase	NATL2A_959226_959912
	*hli11*	HLI protein hli11	NATL2A_967126_966956
		Ferric siderophore transport system, periplasmic binding protein TonB	NATL2A_1209792_1210433
LLI subclade-specific region^*a*^	*hli11*	HLI protein hli11	NATL2A_1052907_1052761
		Endonuclease VIII	NATL2A_1060998_1061843
LLII/III clade-specific region		Helicase, SNF2/RAD54 family	SS120_49788_46600
	*hli*	Phage-encoded HLI protein, HliP	SS120_1097431_1097565
	*hli*	Possible HLI protein	SS120_1097580_1097789
LLII subclade-specific region		DNA double-strand break repair Rad50 ATPase	SS120_645533_642819
		DNA double-strand break repair protein Mre11	SS120_646724_645540
		Possible DNA gyrase/topoisomerase IV, subunit	SS120_647907_647629
HL and LLI shared region^*b*^	*urtE*	Urea ABC transporter, ATPase protein UrtE	NATL2A_1539531_1538824
	*urtD*	Urea ABC transporter, ATPase protein UrtD	NATL2A_1540283_1539531
	*urtC*	Urea ABC transporter, permease protein UrtC	NATL2A_1541395_1540280
	*urtB*	Urea ABC transporter, permease protein UrtB	NATL2A_1542556_1541399
	*urtB*	Urea ABC transporter, substrate binding protein UrtA	NATL2A_1543910_1542633
	*ureG*	Urease accessory protein UreG	NATL2A_1544660_1544043
	*ureF*	Urease accessory protein UreF	NATL2A_1545331_1544660
	*ureE*	Urease accessory protein UreE	NATL2A_1545747_1545352
	*ureD*	Urease accessory protein UreD	NATL2A_1545895_1546818

a XMU1408 does not contain this region.

b MIT9515 does not contain this region.

Our results indicate that the streamlined Prochlorococcus clades share a conserved genomic backbone and have flexible regions that facilitate clade-specific ecological adaptations. In detail, after divergence from the MRCA, the LLII/III clade, which thrives in the deep euphotic zone, might face temporal light shock, whereas the LLI clade might face light shock, nutrient limitations, and DNA damage from UV in the middle to upper euphotic zone. The HL clade, which is well adapted to high-light conditions, might mainly face the problem of nutrient limitations in the oligotrophic surface oceans. These results of clade-specific segment analysis are consistent with a previous finding that associated clade differentiation with gene gain and loss at the whole-genome scale (24). However, our results indicate that genome evolution or clade differentiation is not evenly distributed in the Prochlorococcus genome: most parts of the genome were relatively conserved, but several regions served as evolutionary hotspots, which indicates that genome structure played an important role in Prochlorococcus evolution. Our results also indicate that the ability of the HL and LLI clades to adapt to intermediate- and high-light environments might have been, at least partially, acquired along with genome structure changes after they diverged from the MRCA of all streamlined clades. Thus, our results support the idea that genome streamlining, which resulted in reduced cell volume and increased ability to capture limited available light, might occur in low-light deep water rather than high-light surface water (3). A recent study also found that different environmental conditions (e.g., variation in nutrient chemistry) may influence transcription of transposases and may impact the genomic structure of the cyanobacterium Microcystis aeruginosa (40). Although the exact relations for the various shifts in ecological niches and subsequent adjustments of genome structure are still being determined, our results indicate a mechanism that can drive Prochlorococcus genome evolution.

### Conclusions.

Prochlorococcus has rapidly become the focus of marine microbiology research, since it was discovered 30 years ago, because of its enormous biomass and important role in marine biogeochemical cycles. In this study, we reported genomes of two LLI strains isolated from the western Pacific Ocean and attempted to expand our understanding of Prochlorococcus genome evolution based on genome structure. Using genome rearrangement and backbone analysis, we reconstructed Prochlorococcus phylogeny and found that genome rearrangement coincided with genome reduction and might have played an important role in Prochlorococcus clade differentiation. We also found that the streamlined Prochlorococcus clades shared a conserved genomic backbone, but clade-specific regions facilitated ecological adaptations. Because our study only compared genomes from five cultivated clades that have whole-genome data, there is a large knowledge gap regarding the differentiation of the nonstreamlined LLIV genomes and the MRCA of the highly streamlined clades. In the future, a clearer picture of Prochlorococcus genome evolution will be produced as an increasing number of Prochlorococcus strains are isolated, especially from other LL clades that may have partially streamlined genomes and different genome structures.

## MATERIALS AND METHODS

### Isolation and culture conditions.

Two LLI strains were isolated in the western Pacific Ocean in 2014 (details regarding the isolation locations are shown in Table 1). Briefly, seawater collected with a Niskin bottle was filtered by gravity through two polycarbonate filters with 0.6-μm pore sizes (1), and Pro2 medium stock solution was added to the filtrates (41). These filtrates then were placed in an incubator on board for initial enrichment under constant light flux of 5 to 10 μmol Q m^−2^ s^−1^ at 22°C. After 4 to 8 weeks, the successful initial Prochlorococcus cultures were enriched again by the serial dilution method (42). Briefly, Prochlorococcus cells in the cultures were counted by flow cytometry and then serially diluted into 96-well plates at final concentrations of 1 to 10 cells per well, and a modified ProMM medium (an f/2 vitamin mix instead of the 1× Va vitamin mix) was used. The wells that appeared green were immediately transferred to Pro99 medium and were further confirmed by flow cytometry and 16S-23S rRNA internal transcribed spacer (ITS) sequence analysis. The Prochlorococcus strains were maintained under constant light flux of 10 to 20 μmol Q m^−2^ s^−1^ at 22°C. The presence of heterotrophic bacteria was routinely tested with ProMM (42) and ProAC (43) medium.

### DNA sequencing, assembly, and annotation.

Genomic DNA was collected from 25 ml of the laboratory cultures by centrifugation (10,000 × *g*, 15 min). The QIAamp DNA minikit (Qiagen, Germany) was used to extract genomic DNA. One microgram of genomic DNA was used to construct libraries for Illumina sequencing. Ten nanograms of library DNA was bidirectionally sequenced using a HiSeq 2500 sequencer (Illumina, USA) with a read length of 150 bp. Library construction and sequencing were performed at Shanghai Hanyu Biotechnology Co. (Shanghai, China). Sequencing data of each sample were first trimmed by Trimmomatic v0.32 (44). The clean data with high quality then were assembled using IDBA-UD (45) with a customized kmer (from 21 to 121 with a 10-mer step size). Because the cultures sequenced are not axenic, genome binning was performed using a modified workflow from a well-known method (46) to recover cyanobacterial genomes. Briefly, the binning was based on GC content, sequencing depth, and tetranucleotide frequency to separate each contig by species. To improve the assembly, reads associated with the putative genome bins were extracted for reassembly using SPAdes v3.33.1 (kmer from 21 to 121 with a 10-mer step size) (47) after decreasing the coverage of reads to 100× by BBTools (https://jgi.doe.gov/data-and-tools/bbtools/). Finally, the reassembled bins were estimated by CheckM (35) to assess the completeness and contamination of the recovered genomes. Assembled genomic sequences were annotated using the RAST server against FIGfam, release 70 (48). For comparison, we also reannotated the previously published Prochlorococcus genomes (Table 3) using the same method.

**TABLE 3:** Reference Prochlorococcus genomes used in this study

Strain	Ecotype	Assembly size (bp)	%GC	No. of coding sequences	NCBI accession no.
MIT9515	HLI	1,704,176	30.8	1,964	CP000552
EQPAC1	HLI	1,654,739	30.8	1,958	JNAG00000000
MED4	HLI	1,657,990	30.8	1,962	BX548174
MIT0604	HLII	1,780,061	31.2	2,092	CP007753
AS9601	HLII	1,669,886	31.3	1,938	CP000551
GP2	HLII	1,624,310	31.2	1,878	JNAH00000000
MIT9107	HLII	1,699,937	31	1,994	JNAI00000000
MIT9116	HLII	1,685,398	31	1,984	JNAJ00000000
MIT9123	HLII	1,697,748	31	1,999	JNAK00000000
MIT9201	HLII	1,672,416	31.3	1,987	JNAL00000000
MIT9202	HLII	1,691,453	31.1	2,019	DS999537
MIT9215	HLII	1,738,790	31.1	2,043	CP000825
MIT9301	HLII	1,641,879	31.3	1,927	CP000576
MIT9302	HLII	1,745,343	31.1	2,016	JNAM00000000
MIT9311	HLII	1,711,064	31.2	1,978	JNAN00000000
MIT9312	HLII	1,709,204	31.2	1,979	CP000111
MIT9314	HLII	1,690,556	31.2	1,979	JNAO00000000
MIT9321	HLII	1,658,664	31.2	1,962	JNAP00000000
MIT9322	HLII	1,657,550	31.2	1,961	JNAQ00000000
MIT9401	HLII	1,666,808	31.2	1,969	JNAR00000000
SB	HLII	1,669,823	31.5	1,932	JNAS00000000
MIT0801	LLI	1,929,203	34.9	2,278	CP007754
NATL1A	LLI	1,864,731	35	2,248	CP000553
NATL2A	LLI	1,842,899	35.1	2,209	CP000095
PAC1	LLI	1,841,163	35.1	2,254	JNAX00000000
LG	LLII/III	1,754,063	36.4	1,989	JNAT00000000
MIT0601	LLII/III	1,707,342	37	1,936	JNAU00000000
MIT0602	LLII/III	1,750,918	36.3	2,002	JNAV00000000
MIT0603	LLII/III	1,752,482	36.3	2,012	JNAW00000000
MIT9211	LLII/III	1,688,963	38	1,952	CP000878
SS35	LLII/III	1,751,015	36.4	1,988	JNAZ00000000
SS52	LLII/III	1,754,053	36.4	1,985	JNBE00000000
SS120	LLII/III	1,751,080	36.4	1,982	AE017126
SS2	LLII/III	1,752,772	36.4	1,986	JNAY00000000
SS51	LLII/III	1,746,977	36.4	1,977	JNBD00000000
MIT0701	LLIV	2,592,571	50.6	3,082	JNBA00000000
MIT0702	LLIV	2,583,057	50.6	3,084	JNBB00000000
MIT0703	LLIV	2,575,057	50.6	3,078	JNBC00000000
MIT9303	LLIV	2,682,675	50	3,243	CP000554
MIT9313	LLIV	2,410,873	50.7	3,002	BX548175

### Phylogenomic analysis.

To infer robust phylogenetic relationships between distinct clades, a phylogenetic tree was reconstructed using concatenated protein sequences of 31 core genes (49). Briefly, protein sequences of 31 core genes were concatenated and then aligned with MFFAT v7 (50). Phylogenetic trees were reconstructed using MEGA v7.0 with the maximum likelihood method and 1,000 bootstrap replicates (51). The pan-, core, and accessory genomes of six LLI genomes were calculated using Panseq (52), and the following values for the parameters were set: fragmentation size, 500 bp; homology fragment similarity percent identity cutoff, >70%; blast size, 20.

### Genome rearrangement and backbone analysis.

Genome rearrangement analyses were performed on 14 complete genomes with or without draft genomes. For complete genomes, genome alignment was conducted using the progressiveMauve algorithm in Mauve v2.4.0 (53, 54). Locally colinear block (LCB) detection and backbone analysis were performed using the default parameters in the progressiveMauve algorithm, and a permutation matrix of LCBs was exported for phylogeny reconstruction. MLGO (55, 56) was used to reconstruct the phylogenetic tree based on the permutation matrix of LCBs using maximum likelihood with 1,000 bootstrap replicates. For draft genomes, contigs were first reordered using the Move Contigs tool (57), which reorders all contigs based on whole-genome comparison to the closest complete genome defined by the phylogenomic tree. All contig positions were also manually checked. Accession numbers of all reference genomes used in this study are shown in Table 3.

### Accession number(s).

The genomic sequences of the two newly isolated LLI strains were deposited in GenBank under BioProject number PRJNA474570. The genome accession number of each strain is shown in Table 1.

## Supplementary Material

Supplemental file 1

Supplemental file 2
