# Supplementary material for: Genome Rearrangement Shapes Prochlorococcus Ecological Adaptation
Source: Appl Environ Microbiol. 2018 Aug 17;84(17):e01178-18. doi: 10.1128/AEM.01178-18 (PMC6102989; doi:10.1128/AEM.01178-18)
Supplement: Supplemental file 1 [file zam016188687s1.pdf]

Supplementary material

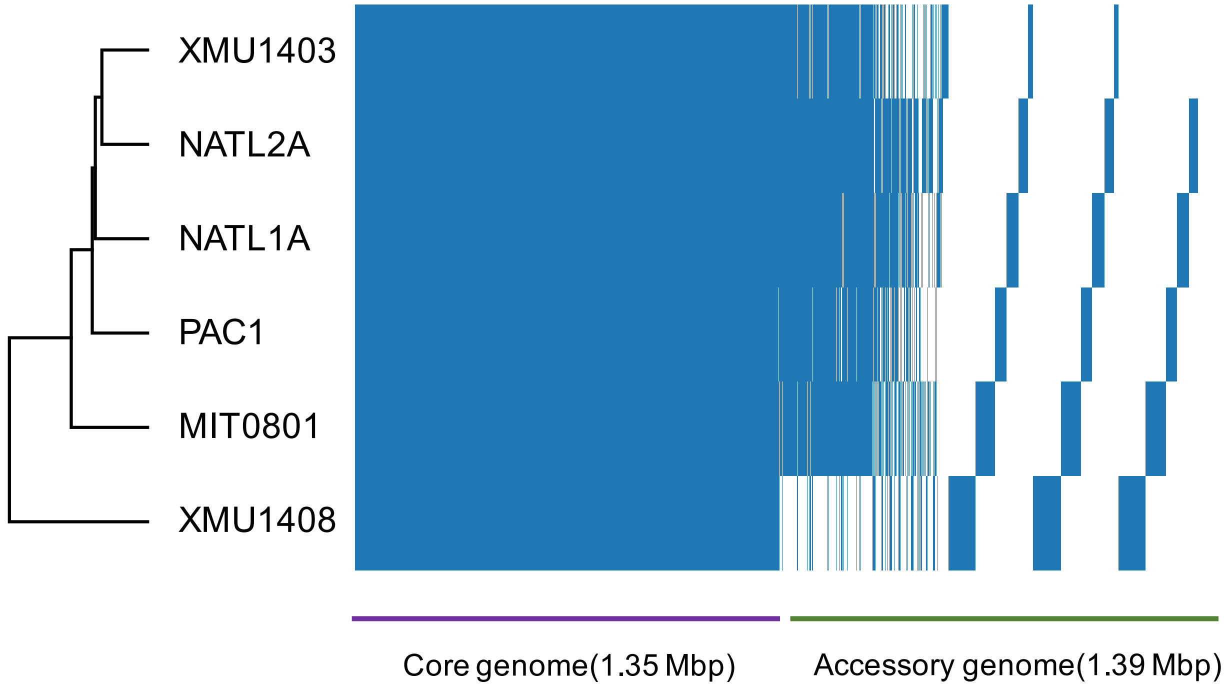

Figure S1. Hierarchical clustering of core/accessory genome data among six *Prochlorococcus* LLI genomes. The accessory genome was generated using the Panseq Core/Accessory Genome Analysis module with fragmentation size of 500 bp and sequence identity cutoff value of 70%. The dendrogram was produced by GENE-E using Euclidean distance and average linkage; blue indicates presence of a locus and white the absence of a locus.

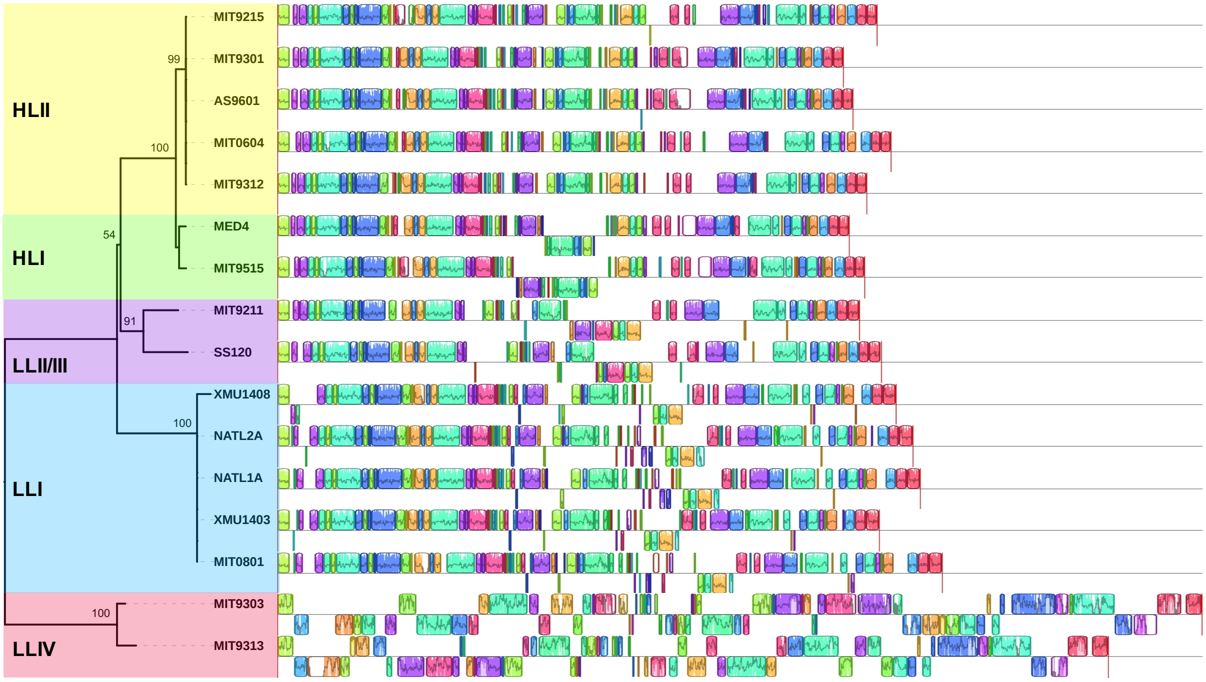

Figure S2. Genome comparison among 16 *Prochlorococcus* genomes. The genome comparison was generated by Mauve (Darling ACE, Mau B, Blattner FR, Perna NT, Genome Research, 14:1394–1403, 2004). Rearrangements of locally collinear blocks (LCBs) among *Prochlorococcus* genomes. LCBs are color-coded. LCBs below the black line have an inverse orientation relative to MIT9301. The large white regions indicate strain-specific content. The phylogenetic tree was reconstructed based on permutations of LCBs with the maximum likelihood method. Numbers at the nodes represent bootstrap values (1000 resamplings). Two draft genomes (XMU1403 and XMU1408) are newly reported in this study.

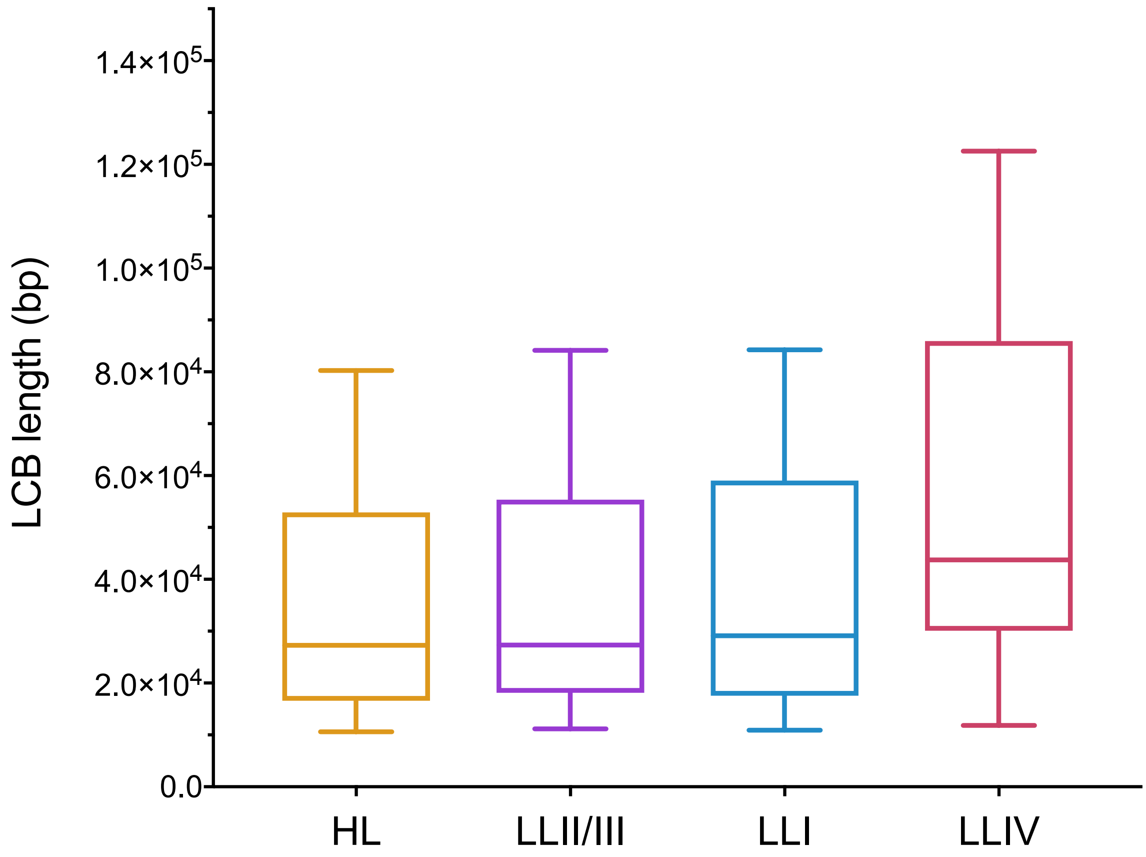

Figure S3. Length comparison of 34 locally collinear blocks (LCBs). The average LCB lengths of the HL, LLI and LLII/III clades are all significantly shorter than that of the LLIV clade ($P<0.001$, $n=34$, one-sample t-test, null hypothesis was set as 55187 bp (the average LCB length of the LLIV clade)). The minimum LCB length used in this comparison is larger than 10 kbp. See more details in Table S1.

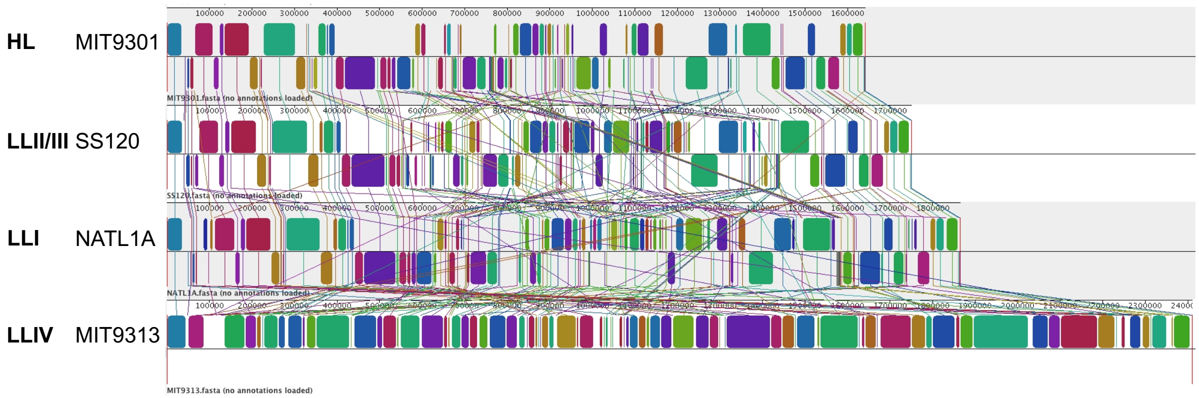

Figure S4. Genome comparison among *Prochlorococcus* genomes. The genome comparison was generated by Mauve (Darling ACE, Mau B, Blattner FR, Perna NT, Genome Research, 14:1394–1403, 2004). Locally collinear blocks (LCBs) are color-coded. LCBs below the black line have an inverse orientation relative to MIT9313. Connecting lines between the two genomes indicate corresponding LCBs.

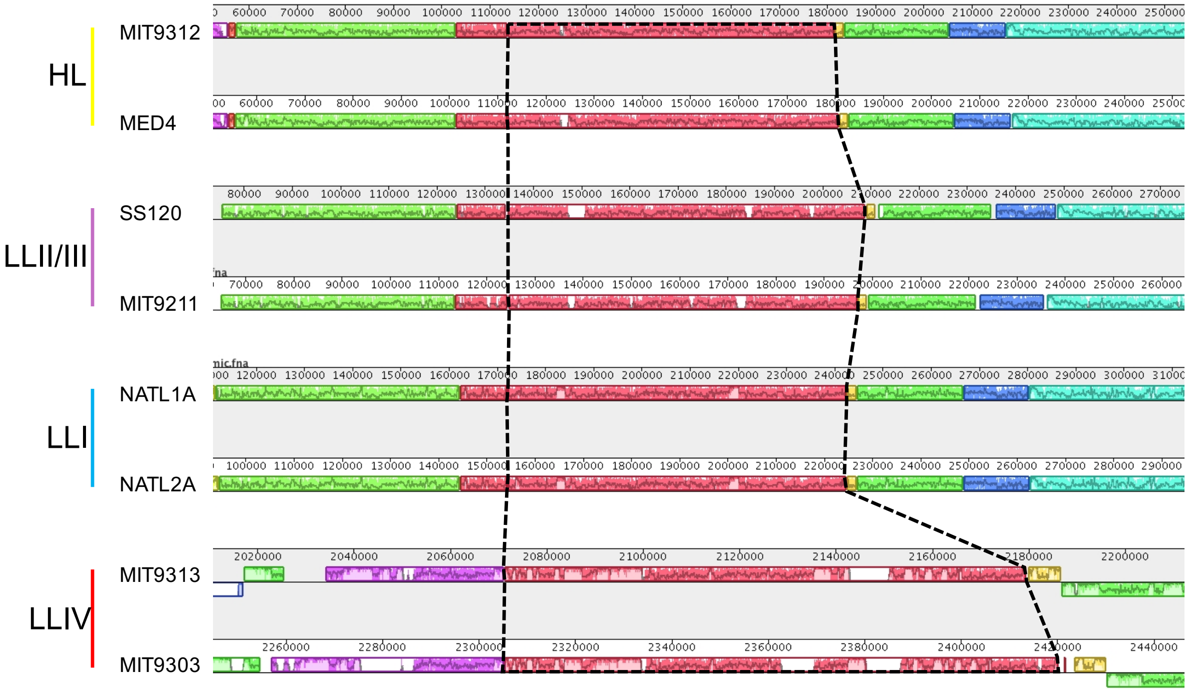

Figure S5. Examples of locally collinear blocks (LCBs) length comparison between different *Prochlorococcus* genomes. The genome comparison was generated by Mauve (Darling ACE, Mau

B, Blattner FR, Perna NT, Genome Research, 14:1394–1403, 2004). Locally collinear blocks (LCBs) are color-coded. LCBs below the black line have an inverse orientation relative to MIT9312. Dash lines between the two genomes indicate corresponding LCBs.

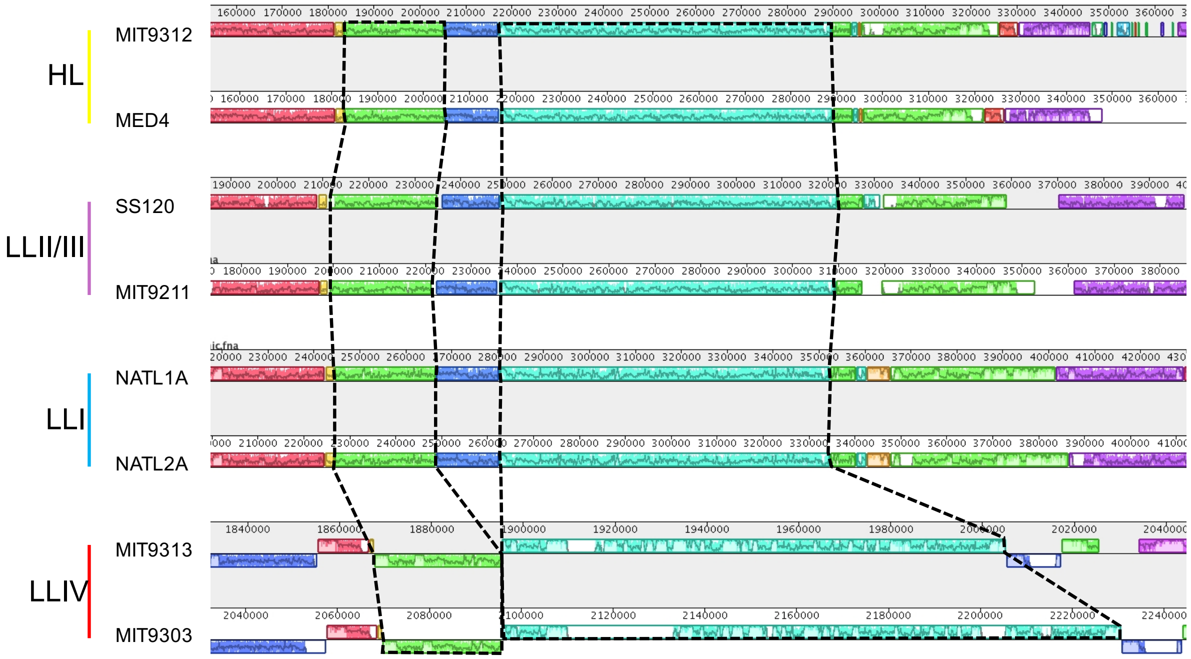

Figure S6. Examples of locally collinear blocks (LCBs) length comparison between different *Prochlorococcus* genomes. The genome comparison was generated by Mauve (Darling ACE, Mau B, Blattner FR, Perna NT, Genome Research, 14:1394–1403, 2004). Locally collinear blocks (LCBs) are color-coded. LCBs below the black line have an inverse orientation relative to MIT9312. Dash lines between the two genomes indicate corresponding LCBs.

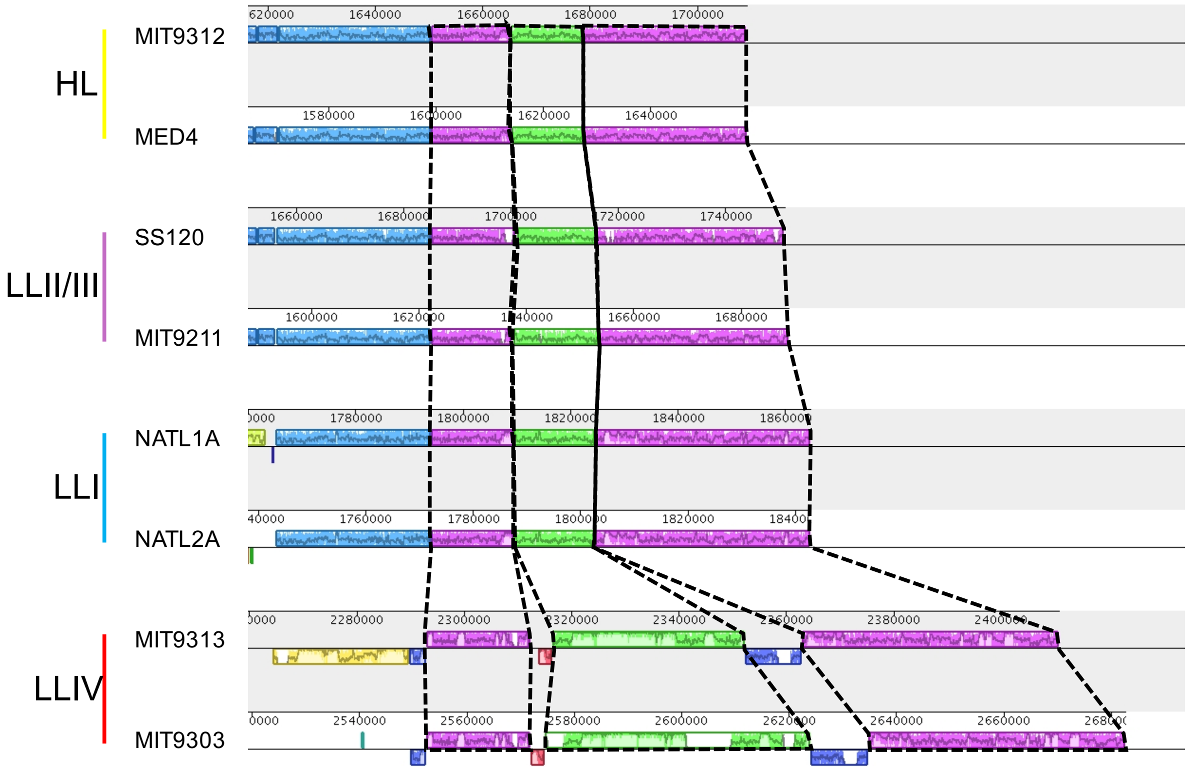

Figure S7. Examples of locally collinear blocks (LCBs) length comparison between different *Prochlorococcus* genomes. The genome comparison was generated by Mauve (Darling ACE, Mau B, Blattner FR, Perna NT, Genome Research, 14:1394–1403, 2004). Locally collinear blocks (LCBs) are color-coded. LCBs below the black line have an inverse orientation relative to MIT9312. Dash lines between the two genomes indicate corresponding LCBs.
